# Non-adherence to anti-tuberculosis treatment and associated factors among TB patients in public health facilities of Hossana town, Southern Ethiopia, 2022

**DOI:** 10.3389/fmed.2024.1360351

**Published:** 2024-03-07

**Authors:** Lire Lemma Tirore, Tadele Ersido, Tilahun Beyene Handiso, Abriham Shiferaw Areba

**Affiliations:** ^1^Department of Public Health, Wachemo University, Hosaina, Ethiopia; ^2^Hossana Town Health Office, Hossana, Ethiopia

**Keywords:** tuberculosis, anti-tuberculosis treatment, non-adherence, infectious diseases, prevalence, associated factors

## Abstract

**Background:**

Non-adherence to anti-tuberculosis treatment is one of the crucial challenges to improving TB treatment outcomes and reducing healthcare costs. The prevalence of non-adherence to anti-tuberculosis treatment is not well documented in the study context. Therefore, this study was aimed at estimating the prevalence of non-adherence to anti-tuberculosis treatment and associated factors among TB patients attending TB clinics in Hosanna town, Southern Ethiopia, in 2022.

**Methods:**

An institution-based cross-sectional study was conducted from April to May 2022. A systematic random sampling technique was employed to select a sample of 233 study subjects from all four public health facilities. According to the order of arrival, every second person was interviewed. Data were collected using a structured questionnaire that was created using several works of literature. A multivariable binary logistic regression analysis was used to identify factors associated with non-adherence to anti-TB drugs. The adjusted odds ratio (AOR) with a 95% confidence interval (CI) was estimated.

**Results:**

The study included 233 tuberculosis (TB) patients with a response rate of 100%. The prevalence of non-adherence was 18% (95% CI: 15.39, 21.82). Being in the continuation phase (AOR = 3.09, 95% CI: 1.16, 8.23), not attending formal education (AOR = 2.47, 95% CI: 1.12, 5.42), not disclosing TB status to their family (AOR = 2.36, 95% CI: 0.11, 5.04) and having poor TB knowledge (AOR = 3.09, 95% CI: 1.48, 6.48) were significantly associated with non-adherence to TB treatment.

**Conclusion:**

Among TB patients, there was a significant prevalence of non-adherence to anti-TB medications. Interventions that target patients with low education status, are in the continuation phase, and do not disclose their TB status to their families are required to improve TB treatment adherence. In addition, improving health education is important to enhance TB knowledge, which has an impact on TB treatment adherence. The need for good drug adherence should be emphasized while counseling TB patients.

## 1 Introduction

The patient’s inability or refusal to take TB medications as directed by the healthcare provider constitutes non-adherence to anti-TB therapy. One of the main challenges to TB control has been recognized as non-adherence to anti-TB therapy. It is the main reason why TB treatment programs fail, and it results in high rates of mortality, MDRTB cases, expensive TB therapy, prolonged infectiousness, and other subpar TB treatment outcomes, as well as the spread of TB in the community. Patients with TB are expected to adhere to treatment plans at a level of more than 90% to facilitate a cure or have a favorable outcome (1, 2).

The World Health Organization (WHO), in its global strategy to eradicate TB, notes that ineffective treatment has led to the emergence of Mycobacterium TB strains that do not respond to treatment with the conventional first-line combination of anti-TB medications, leading to the emergence of multidrug-resistant TB in nearly every nation in the world (3). One of the biggest puzzles and challenges that TB programs encounter is a patient who does not complete TB treatment for any reason (4).

It was recognized that Ethiopia was the first nation in Africa to completely incorporate tuberculosis treatment into its healthcare system (4). Compared to 2015, the nation’s TB incidence and death rate were reduced by 34 and 29%, respectively, in 2020 (5). Ethiopia’s annual TB incidence of 119 cases per 100,000 people in 2021 marks its transition from the list of High Burden Countries (HBCs) in 2021 (6). TB deaths have dropped significantly from 66 per 100,000 population in 2010 to 16 people per 100,000 in 2021. However, TB continues to be the leading infectious killer in Ethiopia (7).

The most recent national TB drug resistance monitoring report estimated that 2.3% of new cases of TB and 17.8% of cases of TB that had already been treated had MDRTB (8). All public-sector facilities in Ethiopia provide free TB treatment services at the primary healthcare level, but the treatment completion rate is still below 90% (9).

Ethiopia has a national guideline for TB management. The standard first-line treatment regimen is given for 6 months in two phases. The first phase is the intensive phase, in which Rifampicin (R), Isoniazid (H), Pyrazinamide (Z), and Ethambutol (E) are given for 2 months. The second phase is the continuation phase, in which Rifampicin (R) and Isoniazid (H) are given for 4 months (10).

A deeper comprehension of the variables influencing TB treatment non-adherence is necessary to improve TB treatment outcomes. Co-morbidities, smoking, alcohol use, accessibility to a healthcare facility, drug side effects, family support, the relationship between the patient and the healthcare provider, the treatment regimen, and sociodemographic factors like age, gender, education level, and income have all been linked to non-adherence to TB treatment (1, 11, 12). Depending on the population’s background characteristics, the association of these factors with non-adherence may vary. To improve TB treatment adherence and produce better results on a national and international basis, various strategies have been investigated. The Directly Observed Therapy Short-course (DOTS) technique for the treatment of TB, as supported by WHO, is the foundation of the Ethiopian National TB Control Program (NTCP) (13, 14). DOTS intervention was more effective in improving treatment completion than self-administration therapy with monthly monitoring. It has successfully improved treatment adherence in patients with TB (15–17).

Despite local, national, and worldwide efforts to prevent and control TB, patients continue to stop taking their medications before they may be declared cured (18). A large percentage of TB patients in sub-Saharan Africa are lost to follow-up, ranging from 11.3 to 29.6% (12). One of the seven nations with poorer treatment success rates is Ethiopia. According to a mapping of TB treatment outcomes in Ethiopia, 5.5% of patients were lost to follow-up, and 0.7% of patients experienced treatment failure. Poor TB treatment outcomes were prevalent overall (9.0%), most likely as a result of non-adherence to therapy and loss of follow-up (1, 2). Southern Nations and Nationalities of Ethiopia had the highest (23.61%) pooled prevalence estimate of non-adherence to anti-TB therapy, according to a systematic review that was conducted to estimate the pooled prevalence of non-adherence to anti-TB treatment in Ethiopia (18). This exceeds the WHO’s recommended level of less than 10% (13, 14). It would be crucial for stakeholders to identify the causes of non-adherence to anti-TB medication to prevent the mortality and morbidity caused by poor adherence to TB treatment. The prevalence of non-adherence to anti-TB treatment is not well documented in the study context. Hence, this study estimated the prevalence of non-adherence to anti-TB treatment and associated factors among TB patients in Hosanna town, southern Ethiopia.

## 2 Materials and methods

### 2.1 Study area and period

The study was carried out in the TB clinics of the public health facilities of Hosanna Town administration, southern Ethiopia, from April to May 2022. The Hadiya zone’s capital, Hosanna Town, is 230 kilometers from Addis Ababa. The town is home to 53 private clinics, 3 public health centers (Bobicho health center, Lich Amba health center, and Hossana health center), 1 private general hospital (Hiwot general hospital), and 1 comprehensive specialized hospital (Nigist Elleni Mohammed Memorial Comprehensive Specialized Hospital). In the public health facilities of Hosanna town, there were 420 TB patients receiving follow-up care. Of these, 162 patients were in the Nigist Elleni Mohammed Memorial Comprehensive Specialized Hospital, 120 were in the Hossana Health Center, 71 were in the Bobicho Health Center, and 67 were in the Lich Amba Health Center. Each health center provides services for more than 250,000 catchment populations, and the comprehensive specialized hospital provides services for more than 3.5 million populations (19).

### 2.2 Study design and population

The institution-based cross-sectional study was carried out. All TB patients at the Hosanna Town TB follow-up clinics who had been on anti-TB medication for at least 1 month were considered as the study population.

### 2.3 Inclusion and exclusion criteria

The study included all patients who had taken anti-TB medication for at least 1 month before the study period (20). Patients with drug-resistant TB (because they need another treatment strategy), severe illness, or those with listening or speaking difficulties were excluded.

### 2.4 Sample size determination

#### 2.4.1 Sample size calculation for the first objective

The sample size was calculated using a single population proportion formula. Assuming a prevalence of non-adherence to TB treatment, *p* = 16.5% from a previous study (21), a 5% margin of error, a 95% confidence level, and a 10% non-response rate, a sample of 233 was considered. The formula is given below:


$$\eta =\frac{{\left(Z_{\alpha /2}\right)}^{2}\,p\,\left(1-p\right)}{d^{2}}$$

Where *n* = sample size required;

*Z* = the critical value corresponding to a 95% confidence level (1.96).

*d* = the desired level of precision (5%).

*p* = the estimated proportion of the non-adherence to TB treatment.


$$n=\frac{{\left(1.96\right)}^{2}*0.165*\left(1-0.835\right)=212}{{\left(0.05\right)}^{2}}$$

The total sample size was 212+21 = 233 when a 10% non-response rate was taken into account (212 × 10% = 21).

#### 2.4.2 Sampling procedures

**FIGURE 1:**
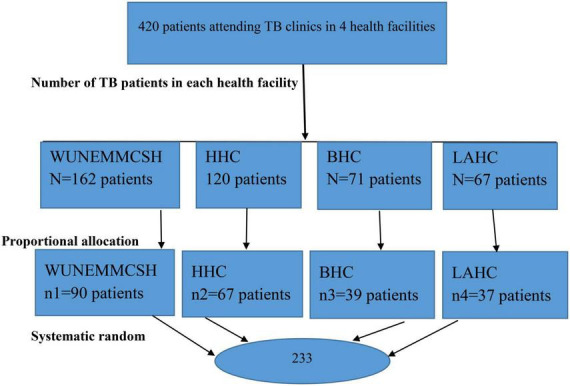
Sample size allocation to study the prevalence of non-Adherence to Anti-TB. Treatment and associated factors among TB patients attending TB clinics in Hosanna Town, Southern Ethiopia 2022. NB, NEMMCSH–Nigist Eleni Mohammed Memorial Compressive Specialized Referral Hospital; HHC, Hosanna Health Center; BHC, Bobicho Health Center; LAHC, Lich Amba Health Center.

A systematic random sampling method was used to select the study subjects. The sampling interval of 2 was considered (*K* = 420/233 = 1.8). The sampling intervals were calculated for each facility and were nearly equal for all facilities (nearly 2 for each). Then, according to the order of arrival, every second person was interviewed until the required sample was achieved. The proportionate allocation to size was made to distribute the sample to all four health facilities (Figure 1).

### 2.5 Study variables

#### 2.5.1 Dependent variable

Non-adherence to anti-TB drugs.

#### 2.5.2 The independent variables were classified as

**Socio-demographic factors** include factors like age (15–30, 31–45, >45), gender (male, female), religion (protestant, orthodox, Muslim, others), ethnicity (Hadiya, Amhara Silte, others), marital status (married, single, divorced, or widowed), educational status (not attended formal education, primary or secondary education, above secondary education), monthly income (<1000, 1000–3000, >3000), and employment (government, housewife, merchant, daily labor, etc.).

**Patient-related factors** include factors like patient knowledge (good or poor), smoking (yes or no), alcohol drinking (yes or no), khat chewing (yes or no), and disclosure status (yes or no).

**Healthcare delivery system-related factors** include factors like distance to the clinic (≤5 , >5 km), time of traveling to the clinic (≤30 min, >30 min), means of transport (foot, vehicles), waiting time (≤ h, >1 h), health information about TB at each visit (yes, no), and fear of stigma and discrimination (yes, no).

**Disease and medication-related factors**: these were taken from the patient follow-up chart and include factors like co-morbid illness (yes, no), drug side effects (yes, no), treatment phase (Intensive Phase, continuation Phase), disease classifications (Pulmonary TB, Extra-Pulmonary TB), treatment regimen (New Patient, Retreatment Patient), and HIV status (Sero-Negative, Sero-Positive, Unspecified).

#### 2.5.3 Operational definitions

**Non-adherence:** A person who scores more than two points on the Adherence Scale is considered to be non-adherent to anti-TB medications. The scale consists of eight items, with the last item having a five-point Likert scale response with options of “never,” “once in a while,” “sometimes,” “usually,” and “always.” The first seven items are scored using the “yes” = 0 and “no” = 1 criterion. According to this Likert scale, values in the range of 0 to 1 were provided at intervals of 0.25, with “0” standing for “never” and “1” for “always.” The level of adherence was assessed using the total score obtained by adding together all of the correct answers, up to a maximum score of 8. The three initial categories of adherence, such as poor, medium, and high, were classified into two categories for data analysis. As a result, poor adherence was classified as non-adherent with a score of more than 2, while high and medium adherence were reclassified as adherent with a score of less than or equal to 2 (22).

**New patients:** patients who had never been treated for TB or had taken it for less than 4 weeks.

**Patient’s knowledge of TB:** Patients’ knowledge about TB was assessed based on their responses to ten questions posed during a face-to-face interview. The assessment was classified as poor or good. Scores of ≥80 and <80%, respectively, were labeled as good and poor knowledge (21).

**Intensive phase:** Patients collect their medication each day from DOTS under supervision for the first 8 weeks of treatment, which is made up of combinations of four drugs (11).

**Continuation phase:** Two months after the start of treatment (following the intensive phase), which consists of a combination of two drugs. The patients collected medication weekly (11).

**Waiting time health facilities** is defined as when TB patients don’t get the required services within optimal time according to BPR standards. This might be either between providers or health facilities. This is greater than 30–60 min during healthcare services (23).

**Co-morbidity:** presence of chronic diseases like HIV and others along with TB.

**The history of smoking and alcohol consumption** has been assessed since the time of starting anti-TB treatment.

**Drug side effects: Drug side effects:** presence of nausea, vomiting, jaundice, skin rash, numbness, and seizures (24).

**Distance of health facilities:** The World Health Organization (WHO) recommends that for optimal access, everyone should live within a 5 km radius of a health facility (2).

**Health information:** It is a provider’s health information to be considered as getting health information about TB at every visit (24).

**Disclosed their TB status with family:** The patients told their family members about their TB status and treatments, gave them attention, and needed moral and financial support (24, 25).

#### 2.5.4 Data collection procedures

To assess non-adherence to anti-TB medications and the factors that determine it, a structured questionnaire was created using several works of literature (13–16, 19). The questionnaire has the following sections: socio-demographic characteristics, disease and medication-related factors, healthcare services and patients’ related factors, knowledge toward TB and anti-TB treatment, and the Adherence Scale. To ensure uniformity, it was first written in English and then translated into Amharic by linguists. Four nurse data collectors were employed. At the TB clinic, when patients came for follow-up care, data was gathered using an interviewer-administered questionnaire in a quiet room. It was collected after verbal informed consent was obtained.

The questionnaire has the following sections: socio-demographic characteristics, Disease and Medication related factors, healthcare services and patients’ related factors, knowledge toward TB and anti-TB treatment and Adherence Scale. To ensure uniformity, it was first written in English and then translated into Amharic by linguists. Four nurse data collectors were employed. At the TB clinic, when patients came for follow-up care, data was gathered using an interviewer-administered questionnaire in a quiet room. It was collected after verbal informed consent was obtained.

#### 2.5.5 Data quality control

For 2 days, data collectors received training on the study’s objectives, the best times to collect data, interviewing procedures, how to approach respondents, and how to maintain confidentiality later. The data collection process was monitored throughout, and daily checks were made to ensure consistency. Any issues that arose were dealt with appropriately.

#### 2.5.6 Data processing and analysis

The statistical program for social sciences (SPSS) version 20 was used to analyze the data after they were entered into EPI INFO version 3.5.3. Tables, graphs, and summary statistics were used to describe the analysis findings. To evaluate the relationship between the dependent variable and the independent factors, multiple logistic regression analysis was utilized. For the multiple binary logistic regression, variables with bivariate analysis *p*-values ≤ 0.25 were taken into consideration. Adjusted odds ratios (AOR) with a 95% confidence interval (CI) were estimated. In the final model, variables with *p*-values less than 0.05 were deemed statistically significant.

## 3 Results

### 3.1 Socio-demographic characteristics of the participants

A total of 233 patients participated in the study, and 100% of them responded. With a mean age of 35.84 (SD = 13.73) years, 109 of them (46.8%) belonged to the 15–30 age range. Males made up more than half (57.5%), and 159 (68.2%) of the participants were married. One hundred two (43.8%) of the study participants attended primary or secondary education, and 112 (43.8%) were merchants. The average monthly income was 2507.89 (± 1765.8) (Table 1).

**TABLE 1:** Socio-demographic characteristics of patients who attended TB clinics at public health institutions of Hosanna town, Southern Ethiopia, 2022.

Variables	Category	Frequency	Percent
Gender	Male	134	57.5
	Female	99	42.5
Age	15–30	109	46.8
	31–45	103	44.2
	>45	21	9
Marital status	Married	159	68.2
	Single	61	26.2
	Divorced or widowed	13	5.6
Educational status	Not attended formal education	59	25.3
	Primary or secondary education	112	43.8
	Above secondary education	72	30.9
Occupation	Government employee	52	22.3
	House wife	36	15.5
	Merchant	114	48.9
	Daily laborer	22	9.4
	Farmers	9	3.9
Monthly income	<1000	58	24.9
	1000–3000	82	35.2
	>3000	93	39.9

### 3.2 Disease and medicine-related factors

Out of 233 individuals, 151 (64.8%) had pulmonary Tb, and 225 (96.6%) were considered new cases. A little over two-thirds (67%) of patients were in the continuation phase of their TB therapy; 45 (19.3%) of them had adverse drug reactions from their TB medications; 194 (83.3%) had tested for HIV; and 19 (8.2%) of them tested positive for HIV (Table 2).

**TABLE 2:** Disease and medicine-related characteristics of the TB patients who attended TB clinics at public health institutions in Hosanna town, Southern Ethiopia, 2022 (*n* = 233).

Variables	Category	Frequency	Percent
Type of TB	Pulmonary Tb	151	64.8
	Extra-pulmonary TB	82	35.2
Treatment regimen	New patient	225	96.6
	Retreatment patient	8	3.4
Treatment phase	Intensive phase	77	33
	Continuation phase	156	67
HIV status	Sero-negative	194	83.3
	Sero-positive	19	8.2
	Unspecified	20	8.6
Experience of drug side effects	Yes	44	18.9
	No	189	81.1
Co-morbidity status	Yes	34	14.6
	No	199	85.4

### 3.3 Health facilities and patient-related factors

More than one-fourth (27.5%) of the participants traveled more than 30 min to pick up their drugs; 139 participants (59.7%) used vehicles as their mode of transportation; and 123 (52.8%) had to pay for transportation to pick up their drugs. One hundred sixty-six (71.2%) of the patients received health information at each visit, 163 (70%) of the participants revealed their TB status to their family members, and nearly one-fourth (23.6%) of the participants were afraid of stigma (Table 3).

**TABLE 3:** Health facility and patient-related factors of the TB patients that attended TB clinics at public health institutions in Hosanna town, Southern Ethiopia, 2022 (*n* = 233).

Variables	Category	Frequency	Percent
Distance of DOTS center	<5 km	219	94
	>5 km	14	6
Traveling time of DOTS center	<30 min	169	72.5
	>30 min	64	27.5
Means of transportation	Foot	94	40.3
	Vehicles	139	59.7
Pay for transport	Yes	123	52.8
	No	110	47.2
Got health information at every visit	Yes	166	71.2
	No	67	28.8
Waiting time at the TB clinic to get services	>1 h	40	17.2
	<1 h	193	82.8
TB status disclosure to the family	Yes	163	70
	No	70	30
Fear of stigma and discrimination	Yes	55	23.6
	No	178	76.4
Smoking	Yes	10	4.3
	No	223	95.7
Use of alcohol	Yes	24	10.3
	No	211	89.7
Knowledge about TB	Good	144	61.8
	Poor	89	38.2

### 3.4 The prevalence of non-adherence to anti-TB medication

The prevalence of non-adherence was 18% (95% CI: 15.39, 21.82).

Among those who did not disclose their TB status to their family, 27.1% were non-adherent to anti-TB medication. The prevalence of non-adherence was 23.1% and 30.5% among those who were in the continuation phase and who had not attended formal education, respectively (Table 4).

**TABLE 4:** Factors associated with non-adherence to anti-TB treatment among TB patients who attended TB clinics at public health institutions in Hosanna town, Southern Ethiopia, 2022 (*n* = 233).

Variables	Category	Non-adherence to anti-TB therapy		COR (95%)	AOR (95% CI)	*p*-value
		**Yes (*n* = 42)**	**No (*n* = 191)**			
Means of transport	Foot	22 (23.4%)	72 (76.6%)	1.82 (0.93, 3.56)	1.18 (0.53, 2.61)	0.688
	Vehicles	20 (14.4%)	119 (85.6%)	1	1	1
Fear of stigma and discrimination	Yes	27 (15.2%)	151 (84.8%)	2.09 (1.02, 4.31)	2.09 (0.45, 9.68)	0.344
	No	15 (27.3%)	40 (72.7%)	1	1	1
Having health information	Yes	25 (15.1%)	141 (84.9%)	1	1	1
	No	17 (25.4%)	50 (74.6%)	2.09 (1.02, 4.31)	2.19 (0.99, 4.86)	0.054
Disclose their TB status to family	Yes	23 (14.1%)	140 (85.9%)	1	1	1
	No	19 (27.1%)	51 (72.9%)	2.27 (1.14, 4.51)	2.36 (1.11, 5.04)	0.026*
Income	<1000 Birr	14 (24.1%)	44 (75.9%)	1.69 (0.73, 3.93)	1.69 (0.65, 4.39)	0.280
	1000–3000	13 (15.9%)	69 (84.1%)	1.66 (0.73,3.74)	1.62 (0.64, 4.10)	0.313
	>3000 Birr	15 (16.1%)	78 (83.9%)	1	1	1
Treatment phase	Intensive phase	6 (7.8%)	71 (92.2%)	1	1	1
	Continuation phase	34 (23.1%)	120 (76.9%)	3.55 (1.43, 8.84)	3.09 (1.16, 8.23)	0.024*
Educational status	No formal education	18 (30.5%)	41 (69.5%)	2.74 (1.36, 5.54)	2.47 (1.12, 5.42)	0.025*
	Formal education	24 (13.8%)	150 (86.2%)	1	1	1
Occupational status	Government employee	3 (5.8%)	49 (94.2%)	1	1	1
	Housewife	31 (22%)	110 (78%)	4.08 (1.01, 16.55)	2.79 (0.59, 13.23)	0.196
	Daily laborer	18 (50%)	18 (50%)	2.03 (0.50, 3.12)	1.50 (0.7, 2.30)	0.56
	Farmers	4 (44.45%)	5 (55.55%)	3.23 (0.50, 1.32)	2.1 (0.32, 2.80)	0.70
	Merchants	50 (43.85%)	64 (56.14%)	0.89 (0.37, 2.12)	1.56 (0.52, 4.66)	0.428
Co-morbidity status	Yes	9 (26.5%)	25 (73.5%)	1.81 (0.78, 4.23)	1.31 (0.47, 3.68)	0.611
	No	33 (16.6%)	166 (83.4%)	1	1	1
Drug side effect	Yes	13 (29.5%)	31 (70.5%)	2.31 (1.08, 4.94)	2.16 (0.92, 5.09)	0.077
	No	29 (15.3%)	160 (84.7%)	1	1	1
Use of Alcohol	Yes	7 (29.2%)	17 (70.8%)	2.05 (0.79, 5.30)	1.79 (0.56, 5.71)	0.325
	No	35 (16.7%)	174 (83.3%)	1	1	1
Knowledge about TB	Good	16 (11.1%)	128 (88.9%)	1	1	1
	Poor	26 (29.2%)	63 (70.8%)	3.30 (1.65, 6.59)	3.09 (1.48, 6.48)	0.003*

Hint: 1: reference category.

*p-value < 0.05.

### 3.5 Factors associated with non-adherence to anti-TB treatment among TB patients

Education level, treatment stage, TB knowledge, and family disclosure status were significantly associated with non-adherence to anti-TB treatment at a *p*-value ≤ 0.05 in multivariable binary logistic regression.

When comparing TB patients in the continuation phase of treatment to those in the intensive phase of treatment, the odds of non-adherence to anti-TB treatment were three times greater for the continuation phase patients (AOR = 3.09, 95% CI: 1.16–8.23). Comparing TB patients who have attended formal school to those who have not, those without formal education had 2.5 times greater odds of not adhering to anti-TB medication (AOR = 2.47, 95% CI: 1.12, 5.42). Participants with poor knowledge of TB and anti-TB therapy had three times greater odds of non-adherence to anti-TB therapy than those with good knowledge of TB and anti-TB therapy (AOR = 3.09, 95% CI: 1.48, 6.48). Comparing TB patients who had not disclosed their TB diagnosis to their family to those who did, the odds of non-adherence to anti-TB treatment were nearly 2.4 times higher among the former (AOR = 2.36, 95% CI 1.11, 5.04) (Table 4).

## 4 Discussion

The objective of this study was to estimate the prevalence of non-adherence to anti-TB treatment and identify factors associated with non-adherence to TB treatment among patients on anti-TB treatment in Hosanna town public health facilities. The prevalence of non-adherence to anti-TB therapy was 18%. This result was consistent with research done in Ethiopia (21, 26, 27) (16.5, 19.5, and 21.2%). This result is less than those of research done in South East Nigeria (12) (24.2%), other studies in Ethiopia (22, 28, 29) (24.7, 24.5, and 55.8%). However, it is higher than studies done in China (25) and northwest Ethiopia (26). This disagreement between studies may be brought about by variations in participant characteristics, the length of time adherence was assessed, and accessibility to healthcare services. For instance, although only TB/HIV-co-infected patients were included in the study in Mekelle, our analysis included both HIV-positive and HIV-negative patients. Patients with a co-infection with HIV are more susceptible to non-adherence. This is due to the fact that the co-infection of HIV and TB causes additional issues with adherence due to the negative effects and high pill burden. Given that patients must receive HIV treatment in addition to TB therapy, they will probably encounter additional difficulties (29, 30).

Multivariable logistic regression analysis revealed that educational status, phase of treatment, poor knowledge, not having to get health information at every visit to TB, and not disclosing TB status to the family were associated with non-adherence to TB treatment.

Patients who were in the continuation phase of treatment had higher odds of non-adherence to anti-TB drugs compared to those who were in the intensive phase. This is in line with the findings of studies done in Ethiopia (24, 27). Because daily therapy was not administered throughout this phase, this may be because the patient believed they were well or that they had recovered from TB. When their TB symptoms disappeared or when they felt better, they may have stopped taking their anti-TB medications. Patients who reported feeling better presumed they had the condition cured (28, 31). The odds of non-adherence were higher among participants who hadn’t attended formal education compared to those who had attended formal education. This finding is supported by studies in Southwest Ethiopia (27), southeast Nigeria (12), Republic of Korea (32), and Indonesia (33). However, in the studies carried out in South Ethiopia (21) and in Plateau State, Nigeria (34) the educational status had no association with non-adherence to anti-TB drugs. This may be because participants who had not received formal education may not have been able to follow directions regarding the treatment provided by the healthcare professionals. They might lack awareness about treatment or the importance of adherence to treatment (33).

The odds of non-adherence were higher among participants who didn’t disclose their TB status to their family compared to those who did disclose their TB status. This finding is supported by studies done in northern Ethiopia (26, 29). Because they feared being discriminated against, patients typically did not disclose their status. One obstacle to TB treatment adherence is a lack of social support (35). Therefore, sharing one’s situation is crucial to receiving various forms of support that can aid in treatment compliance. Poor knowledge about TB and anti-TB treatment had a significant association with non-adherence. Participants who had poor knowledge about TB and anti-TB therapy were 3 times more likely to be non-adherent compared with participants with good knowledge. This is similar to the results of studies in Dawuro zone public healthcare facilities (27), Northwest Ethiopia (26), and Schenzhen, China (25). This could be brought on by patients’ lack of knowledge regarding disease relapse, resistance, and therapy failure. One of the frequent causes of non-adherence is a lack of understanding of TB (36). People with limited information might not be aware of the effects of non-adherence on themselves and their families, so they may view stopping the therapy as an easy option. Better adherence may result from increased disease awareness since the patient is less likely to discontinue medication mid-course (37). Using data collected over the previous month, non-adherence was measured. Participants may therefore experience recall bias. Second, patients were not followed up on, making it impossible to assess their adherence over time. Adherence was solely evaluated by patients reporting missed doses, and patients’ self-reports may have been influenced by the social desirability effect. It is difficult to monitor drug adherence precisely because there is no industry-accepted gold standard for doing so. Additionally, the design of a cross-sectional study does not suggest a causal or temporal association.

## 5 Conclusion

Among TB patients, there was a significant prevalence of non-adherence to anti-TB medications.. Interventions that target patients with low education status, are in the continuation phase, and do not disclose their TB status to their families are required to improve TB treatment adherence. In addition, improving health education is important to enhance TB knowledge, which has an impact on TB treatment adherence. The need for good drug adherence should be emphasized while counseling TB patients.

## Data availability statement

The raw data supporting the conclusions of this article will be made available by the authors, without undue reservation.

## Ethics statement

The studies involving humans were approved by the Institutional Review Board of Wachemo University. The studies were conducted in accordance with the local legislation and institutional requirements. Written informed consent to participate in this study was not required from the participants in accordance with the national legislation and the institutional requirements. The participants provided their verbal informed consent to participate in this study.

## Author contributions

LL: Writing – review and editing, Writing – original draft, Software, Methodology, Investigation, Formal Analysis, Data curation, Conceptualization. TE: Writing – review and editing, Visualization, Validation, Software, Methodology. TB: Writing – review and editing, Visualization, Validation, Software, Methodology. AS: Writing – review and editing, Methodology, Formal Analysis.

## References

[1] Castelnuovo B. Review of compliance with anti-tuberculosis treatment and risk factors for defaulting on treatment in Sub-Saharan Africa. *Afr Health Sci.* (2010) 10:320–4. 21416032 PMC3052808

[2] World Health Organization. *Guidelines for treatment of drug-susceptible tuberculosis and patient care.* Geneva: World Health Organization (2017).

[3] Tiberi S Petersen E Maeurer M Ntoumi F Yeboa-Manu D Mwaba P Taking forward the stop TB partnership and world health organization joint theme for world TB day on march 24th, 2018–“wanted: Leaders for a TB-free world. You can make history. End TB”. *Int J Infect Dis.* (2018) 68:122–4. 10.1016/j.ijid.2018.03.002 29571578

[4] Saravanan M Niguse S Abdulkader M Tsegay E Hailekiros H Gebrekidan A A review of the emergence of drug-resistant tuberculosis (MDR and XDR-TB) and its molecular diagnosis in Ethiopia. *Microb Pathog.* (2018) 117:237–42. 10.1016/j.micpath.2018.02.047 29486274

[5] World Health Organization. *Global TB report.* Geneva: World Health Organization (2021).

[6] Ministry of Health. *Annual performance R, 2014 EFY/2021–2022.* Addis Ababa: Ministry of Health (2022).

[7] Chakaya J Petersen E Nantanda R Mungai B Migliori G Amanullah F The WHO global tuberculosis 2021 report–not so good news and turning the tide back to end TB. *Int J Infect Dis.* (2022) 124:S26–9. 10.1016/j.ijid.2022.03.011 35321845 PMC8934249

[8] World Health Organization. *WHO report 2011: Global tuberculosis control.* Geneva: World Health Organization (2011). 246 p.

[9] Wei M Yongjie Z Zhuoyu Q Biao Y Xi J Wei J Pneumonia is caused by *Mycobacterium tuberculosis*. *Microbes Infect.* (2020) 22:278–84. 32561408 10.1016/j.micinf.2020.05.020 PMC7297158

[10] FMoH. *Guidelines for managing tuberculosis. DR-TB AND LEPROSY IN ETHIOPIA.* 6TH ed. Addis Ababa: FMoH (2017).

[11] Marahatta S Yadav R Giri D Lama S Rijal K Mishra S Barriers to access, diagnosis, and treatment completion for tuberculosis patients in central and western Nepal: A qualitative study among patients, community members, and health care workers. *PLoS One.* (2020) 15:e0227293. 10.1371/journal.pone.0227293 31940375 PMC6961875

[12] Ubajaka C Azuike E Ugoji J Nwibo O Ejiofor O Modebe I Adherence to drug medications amongst tuberculosis patients in a tertiary health institution in south-east Nigeria. *Int J Clin Med.* (2015) 6:399–406.

[13] Harju K. Between donor interest, global models, and local conditions: Treatment and decision-making in the Somalia-Finland tuberculosis control project, 1981–3. *Med Hist.* (2020) 64:94–115. 10.1017/mdh.2019.78 31933504 PMC6945207

[14] Subbaraman R de Mondesert L Musiimenta A Pai M Mayer K Thomas B Digital adherence technologies for the management of tuberculosis therapy: Mapping the landscape and research priorities. *BMJ Glob Health.* (2018) 3:e001018. 10.1136/bmjgh-2018-001018 30364330 PMC6195152

[15] Chaulk C Kazandjian V. Directly observed therapy for treatment completion of pulmonary tuberculosis: Consensus statement of the public health tuberculosis guidelines panel. *JAMA.* (1998) 279:943–8. 10.1001/jama.279.12.943 9544769

[16] Cox H Morrow M Deutschmann P. Long-term efficacy of DOTS regimens for tuberculosis: A systematic review. *BMJ.* (2008) 336:484–7. 10.1136/bmj.39463.640787.BE 18250104 PMC2258398

[17] Pradipta I Houtsma D van Boven J Alffenaar J Hak E. Interventions to improve medication adherence in tuberculosis patients: A systematic review of randomized controlled studies. *NPJ Prim Care Respir Med.* (2020) 30:21. 10.1038/s41533-020-0179-x PMC7214451 32393736

[18] Zegeye A Dessie G Wagnew F Gebrie A Islam S Tesfaye B Prevalence and determinants of anti-tuberculosis treatment non-adherence in Ethiopia: A systematic review and meta-analysis. *PLoS One.* (2019) 14:e0210422. 10.1371/journal.pone.0210422 30629684 PMC6328265

[19] FMoH. *Ethiopian health tiers and referral structure.* Addis Ababa: FMoH (2015).

[20] Tesfahuneygn G Medhin G Legesse M. Adherence to anti-tuberculosis treatment and treatment outcomes among tuberculosis patients in Alamata district, northeast Ethiopia. *BMC Res Notes.* (2015) 8:503. 10.1186/s13104-015-1452-x 26420164 PMC4588463

[21] Ajema D Shibru T Endalew T Gebeyehu S. Level of and associated factors for non-adherence to anti-tuberculosis treatment among tuberculosis patients in Gamo Gofa zone, southern Ethiopia: A cross-sectional study. *BMC Public Health.* (2020) 20:1705. 10.1186/s12889-020-09827-7 33187496 PMC7666453

[22] Gube A Debalkie M Seid K Bisete K Mengesha A Zeynu A Assessment of anti-TB drug nonadherence and associated factors among TB patients attending TB clinics in Arba Minch governmental health institutions, Southern Ethiopia. *Tuberc Res Treat.* (2018) 2018:3705812. 10.1155/2018/3705812 29670768 PMC5835254

[23] Dheda K Gumbo T Maartens G Dooley K McNerney R Murray M The epidemiology, pathogenesis, transmission, diagnosis, and management of multidrug-resistant, extensively drug-resistant, and incurable tuberculosis. *Lancet Respir Med.* (2017) 5:291–360. 10.1016/S2213-2600(17)30079-6 28344011

[24] Tola H Garmaroudi G Shojaeizadeh D Tol A Yekaninejad M Ejeta L The effect of psychosocial factors and patients’ perceptions of tuberculosis treatment non-adherence in Addis Ababa, Ethiopia. *Ethiop J Health Sci.* (2017) 27:447–8. 10.4314/ejhs.v27i5.2 29217949 PMC5615005

[25] Fang X Shen H Hu W Xu Q Jun L Zhang Z Prevalence of and factors influencing anti-tuberculosis treatment non-adherence among patients with pulmonary tuberculosis: A cross-sectional study in Anhui Province, Eastern China. *Med Sci Monit.* (2019) 25:1928. 10.12659/MSM.913510 30869079 PMC6429981

[26] Mekonnen H Azagew A. Non-adherence to anti-tuberculosis treatment, reasons, and associated factors among TB patients attending Gondar town health centers, Northwest Ethiopia. *BMC Res Notes.* (2018) 11:691. 10.1186/s13104-018-3789-4 30285907 PMC6167840

[27] Woimo T Yimer W Bati T Gesesew H. The prevalence and factors associated with anti-tuberculosis treatment non-adherence among pulmonary tuberculosis patients in public health care facilities in South Ethiopia: A cross-sectional study. *BMC Public Health.* (2017) 17:269. 10.1186/s12889-017-4188-9 28320351 PMC5359861

[28] Boru C Shimels T Bilal A. Factors contributing to non-adherence to treatment among TB patients in Sodo Woreda, Gurage Zone, Southern Ethiopia: A qualitative study. *J Infect Public Health.* (2017) 10:527–33. 10.1016/j.jiph.2016.11.018 28189508

[29] Eticha T Kassa E. Non-adherence to anti-TB drugs and its predictors among TB/HIV co-infected patients in Mekelle, Ethiopia. *J Bioanal Biomed.* (2014) 6:61–4.

[30] WHO. *TB/HIV, MDR/XDR-TB, and other challenges.* Geneva: WHO (2008).

[31] Sardar P Jha A Roy D Roy S Guha P Bandyopadhyay D. Intensive-phase non-compliance with anti-tubercular treatment in patients with HIV-TB coinfection: A hospital-based cross-sectional study. *J Commun Health.* (2010) 35:471–8. 10.1007/s10900-009-9215-z 20041282

[32] Choi H Chung H Muntaner C Lee M Kim Y Barry C The impact of social conditions on patient adherence to pulmonary tuberculosis treatment. *Int J Tuberc Lung Dis.* (2016) 20:948–54. 27287649 10.5588/ijtld.15.0759 PMC6013067

[33] Lahdji A Anggraini M Raynalda A. Education level and economic status in increasing adherence to medication in pulmonary tuberculosis patients. *Proc Int Semin Commun Health Med Sci.* (2022) 15:75–6.

[34] Ibrahim L Hadejia I Nguku P Dankoli R Waziri N Akhimien M Factors associated with interruption of treatment among pulmonary tuberculosis patients in Plateau State, Nigeria. 2011. *Pan Afr Med J.* (2014) 17:78. 10.11604/pamj.2014.17.78.3464 24711884 PMC3972906

[35] Gebreweld F Kifle M Gebremicheal F Simel L Gezae M Ghebreyesus S Factors influencing adherence to tuberculosis treatment in Asmara, Eritrea: A qualitative study. *J Health Popul Nutr.* (2018) 37:1. 10.1186/s41043-017-0132-y 29304840 PMC5756387

[36] Chebet N Kirui J Otieno G Sanga D Wanjau G Yoos A. Tuberculosis treatment adherence among patients taking anti-TB drugs in Kilifi County, Kenya. *Afr J Health Sci.* (2022) 35:210–23.

[37] Herrero M Ramos S Arrossi S. Determinants of non-adherence to tuberculosis treatment in Argentina: Barriers related to access to treatment. *Rev Brasil Epidemiol.* (2015) 18:287–98. 10.1590/1980-5497201500020001 26083503

